# A Naturally-Occurring Histone Acetyltransferase Inhibitor Derived from *Garcinia indica* Impairs Newly Acquired and Reactivated Fear Memories

**DOI:** 10.1371/journal.pone.0054463

**Published:** 2013-01-21

**Authors:** Stephanie A. Maddox, Casey S. Watts, Valérie Doyère, Glenn E. Schafe

**Affiliations:** 1 Department of Psychology, Yale University, New Haven, Connecticut, United States of America; 2 Interdepartmental Neuroscience Program, Yale University, New Haven, Connecticut, United States of America; 3 Université Paris-Sud, Centre de Neurosciences Paris-Sud, UMR 8195, Orsay, France; 4 CNRS, Orsay, France; Florida State University, United States of America

## Abstract

The study of the cellular and molecular mechanisms underlying the consolidation and reconsolidation of traumatic fear memories has progressed rapidly in recent years, yet few compounds have emerged that are readily useful in a clinical setting for the treatment of anxiety disorders such as post-traumatic stress disorder (PTSD). Here, we use a combination of biochemical, behavioral, and neurophysiological methods to systematically investigate the ability of garcinol, a naturally-occurring histone acetyltransferase (HAT) inhibitor derived from the rind of the fruit of the Kokum tree (*Garcina indica*), to disrupt the consolidation and reconsolidation of Pavlovian fear conditioning, a widely studied rodent model of PTSD. We show that local infusion of garcinol into the rat lateral amygdala (LA) impairs the training and retrieval-related acetylation of histone H3 in the LA. Further, we show that either intra-LA or systemic administration of garcinol within a narrow window after either fear conditioning or fear memory retrieval significantly impairs the consolidation and reconsolidation of a Pavlovian fear memory and associated neural plasticity in the LA. Our findings suggest that a naturally-occurring compound derived from the diet that regulates chromatin function may be useful in the treatment of newly acquired or recently reactivated traumatic memories.

## Introduction

Newly acquired memories are thought to be inherently unstable, acquiring stability over time as they are ‘consolidated’ into long-term representations in the brain [1]. Later memory retrieval is known to trigger a new phase of instability for a brief window of time during which the memory may be updated (e.g. strengthened or weakened) prior to being re-stabilized in a process known as ‘reconsolidation’ [2], [3]. This window of lability for both consolidation and reconsolidation has attracted considerable experimental attention, fueled in part by the promise of discovering novel therapeutic and/or pharmacological approaches for the treatment of psychiatric disorders ranging from post-traumatic stress disorder (PTSD) to drug addiction that are characterized by unusually strong and persistent memories [4], [5].

The study of the neural and molecular mechanisms underlying the consolidation and reconsolidation of Pavlovian fear conditioning, an animal model of PTSD, has progressed rapidly in recent years [5]–[10]. With notable exceptions [7], findings have collectively suggested that fear memory consolidation and reconsolidation share many of their core molecular features in common, including NMDA-receptor driven activation of protein kinase signaling cascades [11]–[16], the involvement of transcription factors [17], [18], *de novo* mRNA and protein synthesis [19]–[22], and the involvement of immediate early genes [23]–[27]. However, the majority of the pharmacological agents that have been used to disrupt fear memory consolidation and/or reconsolidation in animal models, including mRNA and protein synthesis inhibitors [19], [20], [28], [29], antisense oligonucleotides [23]–[25], and viral vectors [17], [27], [30], are not readily applicable in humans due to potential issues with drug delivery and toxicity. The β-adrenergic antagonist propranolol is an exception to this rule and has received considerable experimental attention for its ability to impair both newly formed and reactivated fear memories in preclinical studies [31]–[33]. However, propranolol has not been shown to be effective in every study [34], and its effectiveness in treating symptoms of PTSD in humans has yielded mixed results [35]–[39]. It is thus of considerable interest to investigate the efficacy of other compounds that are similarly suitable for human consumption which may be used either alone or in combination with existing methods during the lability window to attenuate fearful or traumatic memories.

In recent years, interest has turned toward the examination of a relatively new class of pharmacological agents that target so-called ‘epigenetic’ processes in the treatment of neuropsychiatric disorders [40]–[42]. Epigenetic modifications, including alterations in chromatin structure and DNA methylation, have been widely implicated in memory and cognition [41], [43]–[45]. Chromatin, which consists of DNA packaged tightly around a core of eight histones, is known to be dynamically regulated by acetylation of histones via histone acetyltransferases (HATs). Acetylation causes chromatin structure to relax, leading to enhanced transcription, a process that is readily reversible via a second family of chromatin modifying enzymes known as histone deacetylases (HDACs) [46]–[48]. In a clinical context, studies have suggested that enhancing histone acetylation through HDAC inhibition can rescue the memory deficits associated with cognitive disorders ranging from certain forms of intellectual disabilities to Alzheimer's disease [49]–[53]. However, while enhancing histone acetylation has shown promise for treating neuropsychiatric disorders characterized by memory impairment, traumatic fear memories are an example of a memory-related psychiatric disorder in which it is desirable to impair, rather than enhance, the memory trace.

In the present study, we explore the potential efficacy of a relatively novel and naturally-occurring HAT inhibitor known as garcinol [54], [55], derived from the rind of the fruit of the Kokum tree (*Garcinia indica*), in the treatment of newly formed and reactivated fear memories. We show that garcinol impairs histone acetylation in the lateral nucleus of the amygdala (LA) associated with fear conditioning and retrieval of a fear memory. Further, we show that intra-LA or systemic administration of garcinol within a narrow time window after fear conditioning or fear memory retrieval impairs the consolidation and reconsolidation of a fear memory in a time-limited and retrieval-specific manner. Collectively, our findings suggest the intriguing possibility that a naturally-occurring compound derived from the diet may be useful in the treatment of newly acquired or recently reactivated traumatic memories.

## Results

### Local infusion of garcinol into the amygdala shortly after fear conditioning impairs the consolidation of a fear memory

**Figure 1:**
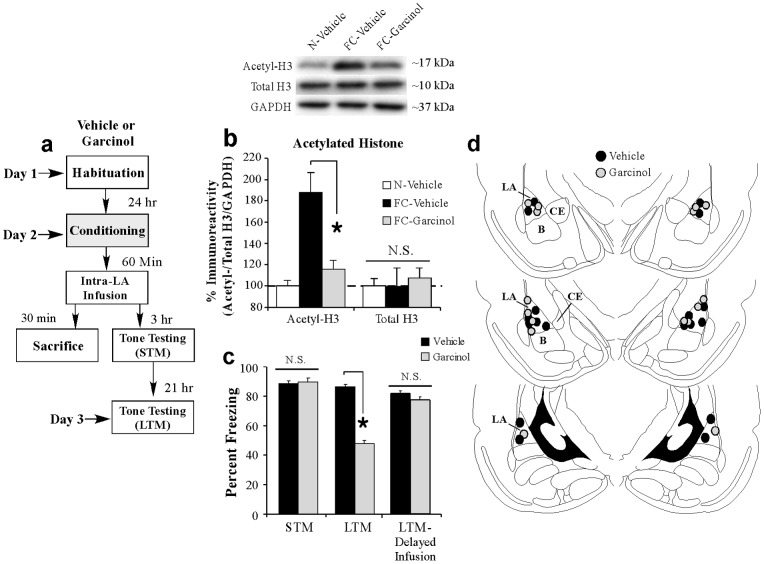
Intra-LA infusions of garcinol impair training-related acetylation of histone H3 and fear memory consolidation. (a) Schematic of the behavioral protocol. Rats were fear conditioned with three tone-shock pairings followed 1 hr later by intra-LA infusion of either vehicle (*n* = 7) or garcinol (500 ng/side; *n* = 7) and were sacrificed 30 min later. A third group did not receive conditioning and was infused with vehicle prior to sacrifice (*n* = 7). Separate groups of rats were fear conditioned with three tone-shock pairings followed 1 hr later by intra-LA infusion of either vehicle (*n* = 9) or garcinol (500 ng/side; *n* = 8) and tested for STM and LTM 3 and 21 hrs later, respectively. (b) Western blot analysis of acetylated and total (non-acetylated) histone H3 from LA homogenates from naïve (N)-Vehicle, fear conditioned (FC)-Vehicle and FC-Garcinol groups. * p<0.05 relative to FC-vehicle and N-Vehicle groups. Representative Western blots are depicted in the inset. (c) Mean (± SEM) percent freezing during the STM and LTM tests in vehicle and garcinol-infused groups. A third group is depicted that received infusion of either vehicle (*n* = 8) or garcinol (*n* = 6) 6 hrs following fear conditioning (‘delayed infusion’) followed by a LTM test 21 hrs later. (d) Cannula placements for rats infused with either vehicle (black circles) or garcinol (gray circles). *p<0.05 relative to vehicle-infused controls.

In our first series of experiments, we asked whether local infusion of garcinol into the LA, the presumed locus of fear memory acquisition and storage [8], [10], [56], , can impair the consolidation of a fear memory. In our first experiment, rats were fear conditioned with three pairings of a tone (conditioned stimulus; CS) with footshock (unconditioned stimulus; US) followed 1 hr later by intra-LA infusion of either vehicle (0.5 µl/side) or garcinol (500 ng/side; 0.5 µl). A portion of the rats was sacrificed 30 min later (90 min after training [58]) to examine the effect of garcinol on the training-related acetylation of histone H3 in the LA (Figure 1a). The remaining rats received tests of short-term memory (STM) and long-term memory (LTM) in a distinct chamber at 3 hr and 21 hr following infusion, respectively (Figure 1a).

Western blotting revealed that infusion of garcinol following auditory fear conditioning significantly impaired the training-related acetylation of histone H3 in the LA [*F*_(2,18)_  = 15.3, *p*<0.05; Figure 1b]. Duncan's post-hoc t-tests revealed that the fear conditioned (FC)-Garcinol group exhibited significantly lower levels of H3 acetylation relative to the FC-Vehicle group (*p*<0.05) that did not differ significantly from naïve (N)-Vehicle controls (*p*>0.05). Importantly, no differences were observed in total (non-acetylated) levels of histone H3 [*F*_(2,18)_ = 0.14; Figure 1b] or in the loading protein GAPDH [*F*_(2,18)_ = 0.33; data not shown].

In our behavioral experiments, vehicle and garcinol-infused rats exhibited equivalent levels freezing during the STM test [*t*_(15)_ = 0.38; Figure 1c], indicating that garcinol has no effect on STM. However, the following day garcinol-treated rats exhibited impaired LTM relative to the vehicle-infused group [*t*_(15)_ = 14.3, *p*<0.01; Figure 1c]. Further, we found that the effect of garcinol on fear memory consolidation is temporally constrained; when rats were given intra-LA infusion of garcinol 6 hr following training there was no effect on LTM [*t*_(12)_ = 1.54; Figure 1c]. Thus, intra-LA infusion of garcinol within a narrow window (1 hr) following Pavlovian fear conditioning can significantly impair the training related acetylation of histone H3 in the LA and the consolidation of a fear memory.

### Local infusion of garcinol into the amygdala shortly after fear memory retrieval impairs the reconsolidation of a fear memory

**Figure 2:**
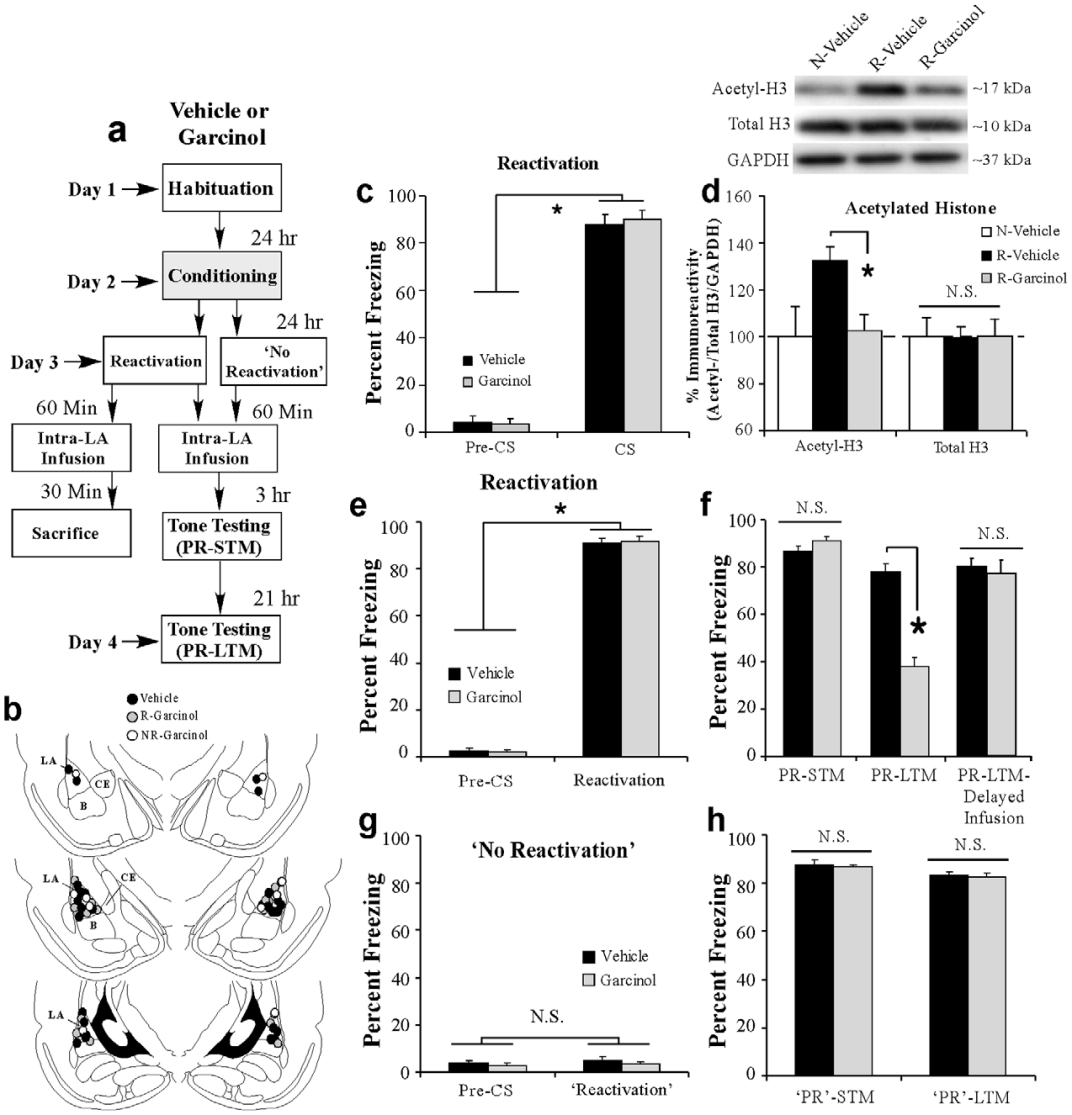
Garcinol impairs retrieval-related acetylation of histone H3 in the LA and fear memory reconsolidation. (a) Schematic of the behavioral protocol. Rats were fear conditioned with three tone-shock pairings. Twenty four hrs following training rats were given a memory reactivation session consisting of a single tone CS presentation followed 1 hr later by intra-LA infusions of vehicle (*n* = 8) or garcinol (500 ng/side; *n* = 7). All rats were sacrificed 30 min following infusion. A third group did not receive conditioning or retrieval testing and was infused with vehicle prior to sacrifice (*n* = 7). Separate groups of rats were fear conditioned followed 24 hr later by a memory reactivation session consisting of a single tone CS presentation followed 1 hr later by intra-LA infusion of vehicle (*n* = 9) or garcinol (500 ng/side; *n* = 8). Two additional groups of rats were given a ‘no-reactivation’ session followed by infusion of vehicle (*n* = 7) or garcinol (500 ng/side; *n* = 5). All rats were then tested for PR-STM and PR-LTM 3 and 21 hrs later, respectively. (b) Cannula placements for rats infused with either vehicle (black circles) or garcinol (gray circles). (c) Memory retrieval data for the Reactivated (R)-Garcinol and R-Vehicle groups used in the Western blotting experiments. *p<0.05 relative to the pre-CS period. (d) Western blot analysis of acetylated and total histone H3 from LA homogenates taken from Naïve (N)-vehicle, R-Vehicle and R-Garcinol groups. * p<0.05 relative to R-Vehicle and N-Vehicle groups. Representative Western blots are depicted in the inset. (e) Memory retrieval data for the Reactivated (R)-Garcinol and R-Vehicle groups in the behavioral experiments. *p<0.05 relative to the pre-CS period. (f) Mean (± SEM) percent freezing during the PR-STM and PR-LTM tests in R-Vehicle and R-Garcinol groups. A third group is depicted that received infusion of either vehicle (*n* = 9) or garcinol (*n* = 6) 6 hrs following retrieval (‘delayed infusion’) followed by a PR-LTM test 21 hrs later. *p<0.05 relative to vehicle-infused controls. (g) Memory retrieval data for the Non-reactivated (NR)-Garcinol and NR-Vehicle groups. (h) Mean (± SEM) percent freezing during the ‘PR’-STM and ‘PR’-LTM tests in NR-Vehicle and NR-Garcinol groups.

In our second series of experiments, we asked whether local infusion of garcinol into the LA shortly after fear memory retrieval can impair the reconsolidation of a fear memory. Rats were fear conditioned as before followed 24 h later by a fear memory retrieval (or ‘reactivation’) session consisting of a single tone CS presentation. One hour following fear memory reactivation, rats received intra-LA infusion of either vehicle (0.5 μl/side) or garcinol (500 ng/side; 0.5 μl). A portion of the rats was sacrificed 30 min later (90 min after retrieval [59]) to examine the effect of garcinol on the retrieval-related acetylation of histone H3 in the LA (Figure 2a). The remaining rats received tests of post-reactivation short-term memory (PR-STM) and post-reactivation long-term memory (PR-LTM) at 3 hr and 21 hr after infusion, respectively (Figure 2a).

In our Western blotting experiments, both vehicle- and garcinol-infused rats exhibited significant and equivalent memory recall during the reactivation session; the ANOVA (group by trial) revealed a significant main effect of trial [pre-CS vs. CS; *F*_(1,13)_ = 555.01, *p*<0.05], but no significant main effect of group [*F*_(1,13)_ = 0.09; Figure 2c]. Further, infusion of garcinol following fear memory reactivation resulted in a significant reduction in the retrieval-related acetylation of histone H3 in the LA [*F*_(2,19)_ = 4.376, *p*<0.05; Figure 2d]. Duncan's post-hoc t-tests revealed that the reactivated (R)-Garcinol group exhibited significantly lower levels of H3 acetylation relative to the R-Vehicle group (*p*<0.05) that did not differ significantly from the naïve (N)-Vehicle group (*p*>0.05). Moreover, no differences were observed in total protein levels of histone H3 [*F*_(2,19)_ = 0.002; Figure 2d] or in the loading protein GAPDH [*F*_(2,19)_ = 0.84; data not shown].

In our behavioral experiments, both vehicle- and garcinol-treated rats exhibited significant and equivalent memory recall during the reactivation session; the ANOVA (group by trial) revealed a significant main effect of trial [pre-CS vs. CS; *F*_(1,15)_ = 2613.88, *p*<0.01], but no significant main effect of group [*F*_(1,15)_ = 0.01; Figure 2e]. Further, both groups exhibited equivalent levels of freezing during the PR-STM test [*t*_(15)_ = 1.59; Figure 2f], indicating that garcinol has no effect on the retention of a fear memory when the animals are tested shortly after memory reactivation and infusion. However, the following day garcinol-treated rats exhibited impaired PR-LTM compared to the vehicle group [*t*_(15)_ = 9.81, *p*<0.01; Figure 2f]. Further, similar to that observed in our consolidation experiments, we found that the effect of garcinol on fear memory reconsolidation is temporally constrained; when rats were given intra-LA infusion of garcinol 6 hrs following memory reactivation there was no effect on PR-LTM [*t*_(13)_ = 0.40 Figure 2f]. Thus, intra-LA infusion of garcinol within a narrow window (1 hr) following fear memory retrieval can significantly impair retrieval-related acetylation of histone H3 in the LA and the reconsolidation of a fear memory.

Importantly, in a separate experiment we observed that the reconsolidation disruption produced by garcinol is specific to a reactivated memory. Rats were fear conditioned as before, followed 24 h later by a ‘no-reactivation’ session in which they were placed in the testing context without a tone presentation. One hour following the ‘no-reactivation’ session, rats received intra-LA infusion of either vehicle (0.5 µl) or garcinol (500 ng/side; 0.5 µl) followed 3 and 21 h later by tests of ‘PR’-STM and ‘PR’-LTM (Figure 2a). Analysis of the reactivation session data revealed that both groups showed equivalently low levels of freezing during the ‘pre-CS’ period and during the 30 sec period when the tone would have been presented during the reactivation session (Figure 2g). An ANOVA (group by trial) revealed no significant effect of group [*F*_(1,10)_ = 0.57] or trial [*F*_(1,10)_ = 0.83]. Similarly, both vehicle and garcinol-treated rats exhibited equivalently high levels of freezing during the ‘PR’-STM test [*t*_(10)_ = 0.37; Figure 2h] and the ‘PR’-LTM test [*t*_(10)_ = 0.53; Figure 2h], indicating that garcinol is only effective at impairing a fear memory in a reconsolidation paradigm if administered around the time of active memory recall.

### Fear memories that fail to reconsolidate following treatment with garcinol are impaired in an enduring manner

**Figure 3:**
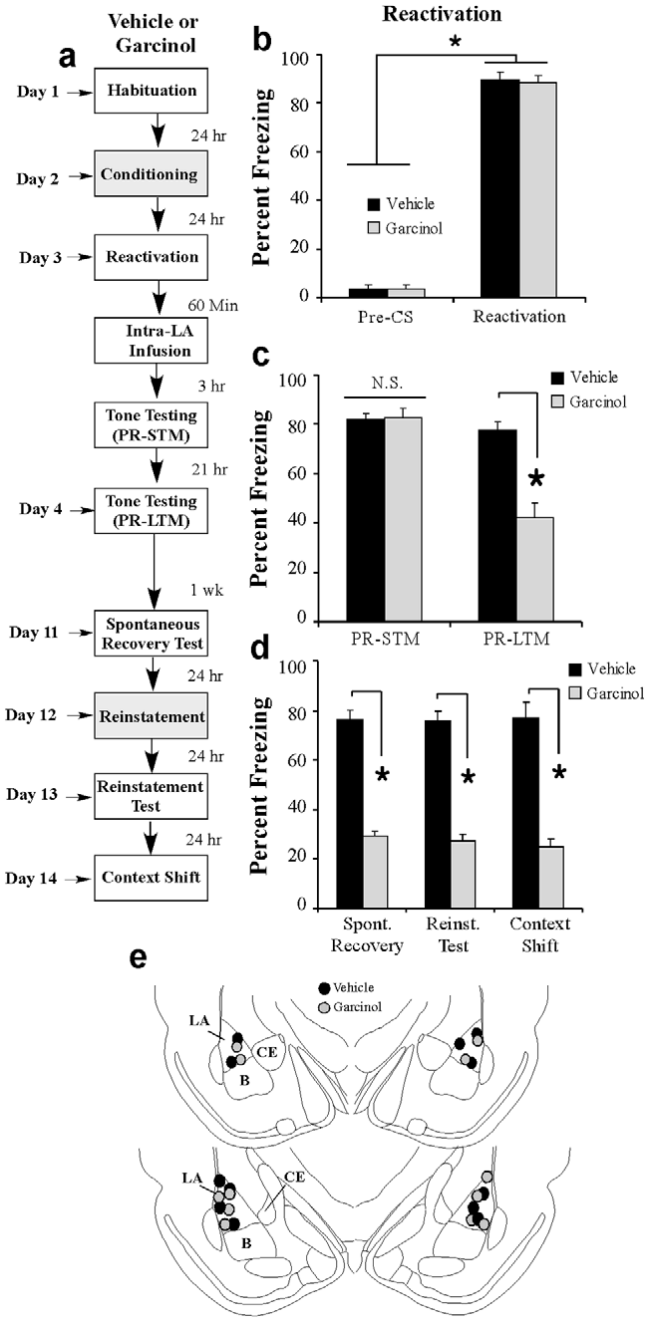
The effect of garcinol on fear memory reconsolidation is not sensitive to spontaneous recovery, reinstatement, or to a shift in testing context. (a) Schematic of the behavioral protocol (see text for details). (b) Memory retrieval data for rats given intra-LA infusion of vehicle (*n* = 6) or garcinol (*n* = 6). *p<0.05 relative to the pre-CS period. (c) Mean (± SEM) percent freezing during the PR-STM and PR-LTM tests in vehicle and garcinol-infused rats. *p<0.05 relative to vehicle-infused controls. (d) Mean (± SEM) percent freezing during the spontaneous recovery, reinstatement, and context shift tests. (e) Cannula placements for rats infused with either vehicle (black circles) or garcinol (gray circles). *p<0.05 relative to vehicle-infused controls.

Our experiments thus far collectively suggest that local infusion of garcinol into the LA impairs reconsolidation of an auditory fear memory in a time-limited and retrieval-specific manner. Previous studies have shown that amygdala-dependent fear memories that are lost due to interference with the reconsolidation process are lost in an enduring manner; they are not sensitive to spontaneous recovery, reinstatement, or renewal in a new testing context [25], [32], [33], [59], [60]. Here, we asked whether the reconsolidation deficit induced by garcinol is similarly insensitive to spontaneous recovery, reinstatement, or to a shift in the testing context. Rats were fear conditioned as before followed 24 h later by a reactivation trial in a distinct context (Chamber B). One hour later, rats were given intra-LA infusion of either vehicle (0.5 μl/side) or garcinol (500 ng/side;0.5 μl) followed 3 and 21 hrs later by tests of PR-STM and PR-LTM in Chamber B. One week later, rats were re-tested for spontaneous recovery of the fear memory in Chamber B. The next day, rats underwent a fear reinstatement session in a novel context (Chamber C) consisting of exposure to three unsignaled footshocks [60] followed 24 h later by a third test of fear memory in Chamber B (Reinstatement Test). Finally, rats were placed in another novel context (Chamber D) and tested with three tone CS presentations to examine whether fear to the tone re-emerges when the animals are tested outside of original reconsolidation testing context (Context Shift) (Figure 3a).

During the original reactivation session, both groups showed equivalently high levels of memory retrieval (Figure 3b); the ANOVA (group by trial) revealed a significant main effect of trial [pre-CS vs. CS; *F*_(1,10)_ = 2790.12, *p*<0.01] but not of group [*F*_(1,10)_ = 0.04]. Further, consistent with our previous experiments, garcinol-treated rats showed intact memory during the PR-STM test [*t*_(10)_ = 0.20], but impaired memory retention during the PR-LTM test [*t*_(10)_ = 5.34, *p*<0.01; Figure 3c]. Importantly, during the test of spontaneous recovery 1 week later, garcinol-treated rats continued to exhibit memory impairment while the vehicle control group exhibited high levels of retention [*t*_(10)_ = 11.33, *p*<0.01; Figure 3d]. During the reinstatement session administered on the next day, both groups exhibited significant post-shock freezing in Chamber C (data not shown). An ANOVA (group by trial) revealed a significant main effect of trial [pre-shock vs. post-shock period; *F*_(3,30)_ = 412.4, *p*<0.01] but not of group [*F*_(1,10)_ = 0.55], indicating an increase in freezing relative to the pre-shock period in both groups. When re-tested 24 hrs later for evidence of reinstatement of fear in Chamber B, however, garcinol-treated rats continued to exhibit memory impairment while the vehicle group exhibited high levels of freezing [*t*_(10)_ = 9.68, *p*<0.01; Figure 3d], suggesting that the garcinol-induced reconsolidation deficit is not sensitive to reinstatement following exposure to an aversive event equivalent in strength to the original aversive experience. Finally, during the context shift test in Chamber D, garcinol-treated rats continued to exhibit memory impairment while the vehicle group exhibited high levels of freezing [*t*_(10)_ = 7.49, *p*<0.01], suggesting that fear memories that are lost following treatment with garcinol in a reconsolidation paradigm do not re-emerge in a different testing context (Figure 3d).

### Garcinol effectively impairs the reconsolidation of an older fear memory

**Figure 4:**
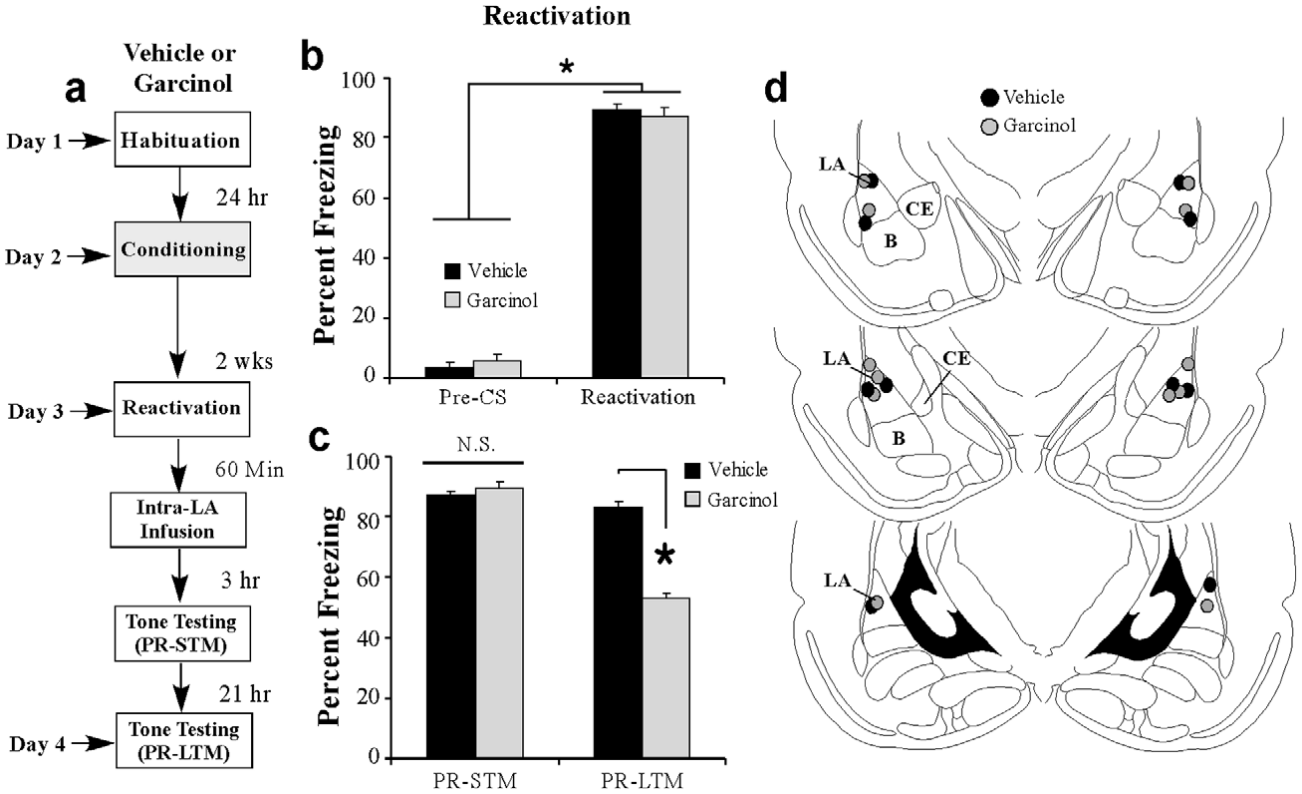
Intra-LA infusion of garcinol impairs the reconsolidation of a ‘well-consolidated’ fear memory. (a) Rats were fear conditioned with three tone-shock pairings. Two weeks following training rats were given a memory reactivation session consisting of a single tone CS presentation followed 1 hr later by intra-LA infusion of vehicle (*n* = 5) or garcinol (500 ng/side; *n* = 6). (b) Memory retrieval data for the vehicle and garcinol-infused groups. *p<0.05 relative to the pre-CS period. (c) Mean (± SEM) percent freezing during the PR-STM and PR-LTM tests in vehicle and garcinol-infused rats. (d) Cannula placements for rats infused with either vehicle (black circles) or garcinol (gray circles). *p<0.05 relative to vehicle-infused controls.

In each of our previous experiments, we reactivated the fear memory within 24 hrs following training. We next asked whether garcinol can impair the reconsolidation of an older, ‘well-consolidated’ memory. Rats were fear conditioned as before followed 2 weeks later by a memory reactivation trial and intra-LA infusion of either vehicle (0.5 μl/side) or garcinol (500 ng/side; 0.5 μl; Figure 4a). Both groups showed equivalently high levels of freezing during the reactivation session (Figure 4b); an ANOVA (group by trial) revealed a significant main effect of trial [pre-CS vs. CS; *F*_(1,9)_ = 1540.99, *p*<0.01] but not of group [*F*_(1,9)_ = 0.10]. Three hours following memory reactivation and drug infusion, both vehicle and garcinol-infused groups displayed equivalent levels of freezing during the PR-STM test [*t*_(9)_ = 0.04; Figure 4c]. On the following day, however, garcinol-treated rats exhibited impaired PR-LTM relative to the vehicle-infused controls [*t*_(9)_ = 10.69, *p*<0.05; Figure 4c]. Thus, even older, ‘well-consolidated’ memories are susceptible to reconsolidation impairment using garcinol.

### Local infusion of garcinol into the amygdala shortly after fear conditioning impairs the consolidation of training-related neural plasticity in the LA

**Figure 5:**
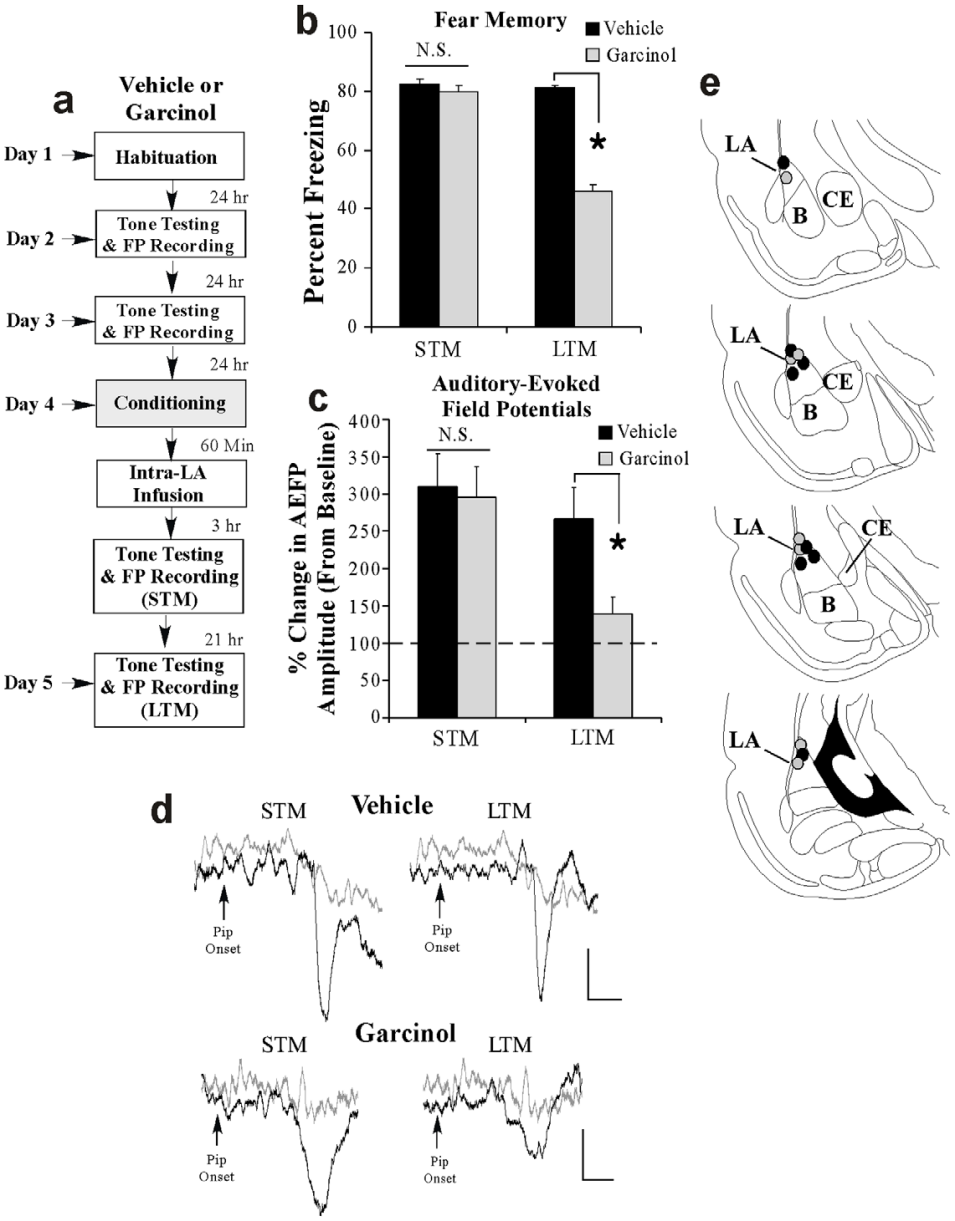
Intra-LA infusion of garcinol impairs fear memory consolidation and the consolidation of training-related neural plasticity in the LA. (a) Rats were given two baseline AEFP recording sessions on separate days followed by fear conditioning with three tone-pip-shock pairings followed 1 hr later by intra-LA infusion of either vehicle (*n* = 8) or garcinol (500 ng/side; *n* = 7). Rats in each group were then tested for STM and LTM 3 and 21 hrs later while AEFPs were recorded from the LA. (b) Mean (± SEM) percent freezing during the STM and LTM tests in vehicle and garcinol-infused groups. (c) Mean (± SEM) percent of change in AEFP amplitude during the STM and LTM tests in vehicle and garcinol-infused rats, relative to baseline. *p<0.05 relative to vehicle-infused controls. (d) Representative AEFPs recorded from the LA for each group during baseline (light gray trace), STM and LTM sessions (darker traces). Scale bar  = 10 µV, 5 ms. (e) Electrode placements for rats infused with either vehicle (black circles) or garcinol (gray circles).

We next asked whether garcinol can impair the consolidation of training-related enhancements in tone-evoked neural activity in the LA, a neurophysiological correlate of fear conditioning [10], [61], [62]. Rats were fear conditioned with 3 pairings of a modified tone CS with footshock (see Methods) followed 1 hr later by intra-LA infusion of vehicle (0.5 µl/side) or garcinol (500 ng/side; 0.5 µl). All rats then received tests of STM and LTM 3 and 21 hr later while auditory-evoked field potentials (AEFPs) were recorded from the LA (Figure 5a). As in our previous experiments, we found that intra-LA infusion garcinol had no effect on STM [*t*_(13)_ = 0.77; Figure 5b] yet significantly impaired LTM [*t*_(13)_ = 14.65, *p*<0.01] relative to vehicle-infused controls (Figure 5b). Similarly, analysis of the neurophysiological data revealed that both vehicle- and garcinol-infused rats exhibited significant enhancements in the amplitude of the short-latency component (∼12–16 ms) of the AEFP in the LA during the STM test relative to baseline [vehicle: *t*_(7)_ = 4.65, *p*<0.05; garcinol: *t*_(6)_ = 6.67, *p*<0.05] that did not differ from each other [*t*_(13)_ = 0.26; Figure 5c]. However, during the LTM test garcinol-treated rats exhibited significantly less AEFP amplitude change relative to vehicle-infused controls [*t*_(13)_ = 2.90, *p*<0.05; Figure 5c]. Thus, intra-LA infusion of garcinol shortly following training can significantly impair, in parallel, both the consolidation of a fear memory and the consolidation of training-related neural plasticity in the LA.

### Local infusion of garcinol into the amygdala shortly after fear memory retrieval impairs memory-related neural plasticity in the LA

**Figure 6:**
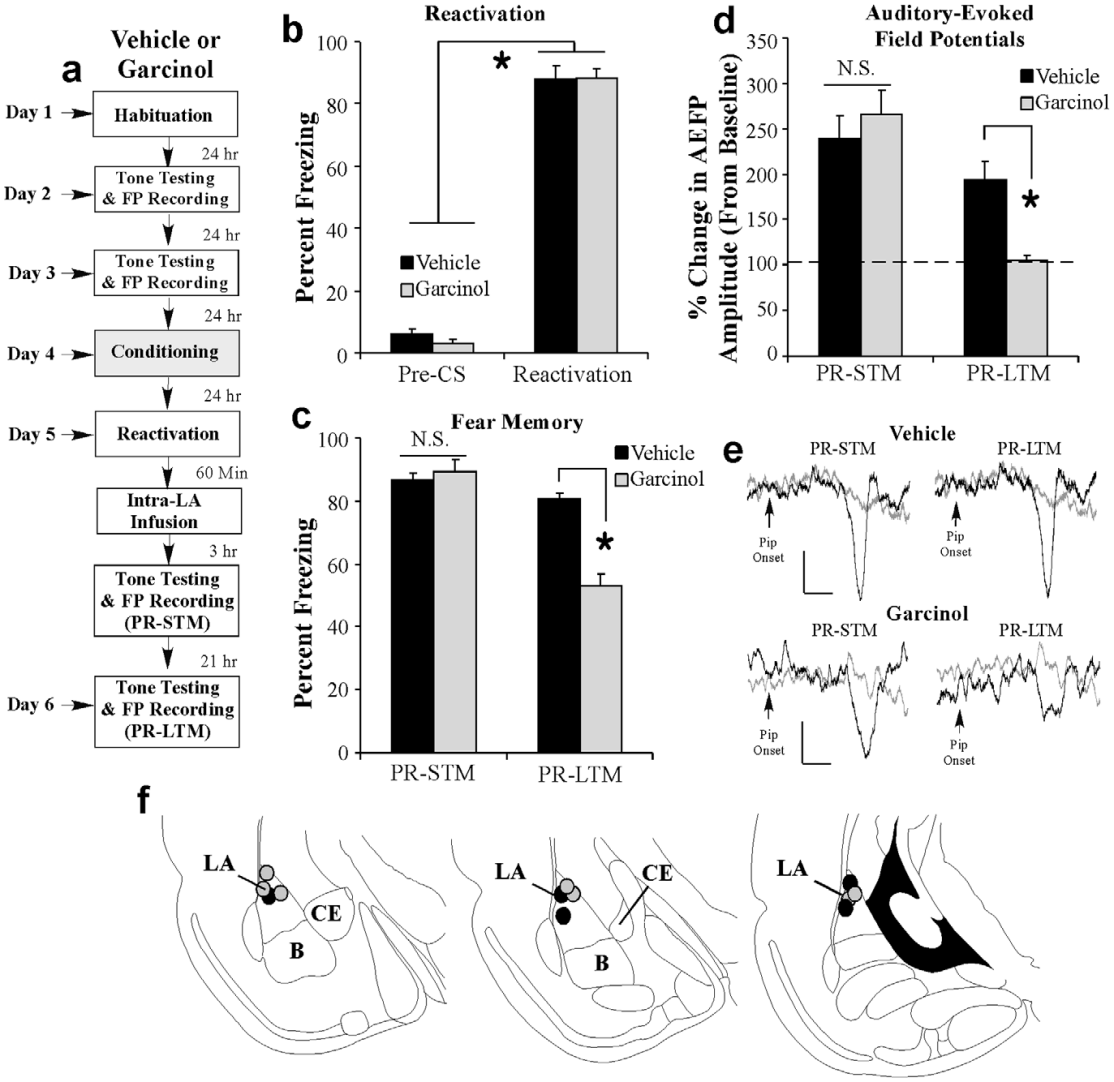
Intra-LA infusion of garcinol impairs fear memory reconsolidation and memory-related neural plasticity in the LA. (a) Rats were given two baseline AEFP recording sessions on separate days followed by fear conditioning with three tone-pip-shock pairings. Twenty four hrs following training rats were given a memory reactivation session consisting of a single tone-pip CS presentation followed 1 hr later by intra-LA infusions of vehicle (*n* = 5) or garcinol (500 ng/side; *n* = 7). Rats in each group were then tested for PR-STM and PR-LTM 3 and 21 hrs later while AEFPs were recorded from the LA. (b) Memory retrieval data for the vehicle and garcinol-infused groups. *p<0.05 relative to the pre-CS period. (c) Mean (± SEM) percent freezing during the PR-STM and PR-LTM tests in vehicle and garcinol-infused groups. (d) Mean (± SEM) percent of change in AEFP amplitude during the PR-STM and PR-LTM tests in vehicle and garcinol-infused rats, relative to baseline. *p<0.05 relative to vehicle-infused controls. (e) Representative AEFPs recorded from the LA for each group during baseline (light gray trace), PR-STM and PR-LTM sessions (darker traces). Scale bar  = 10 µV, 5 ms. (f) Electrode placements for rats infused with either vehicle (black circles) or garcinol (gray circles).

We next examined the effect of post-retrieval administration of garcinol on memory-related neural plasticity in the LA [63]. Rats were fear conditioned as before followed 24 h later by a reactivation session consisting of a single presentation of a modified tone CS (see Methods). One hr following the reactivation session, rats received intra-LA infusion of either vehicle (0.5 µl/side) or garcinol (500 ng/side; 0.5 µl) followed 3 and 21 h later by tests of PR-STM and PR-LTM while AEFPs were recorded from the LA (Figure 6a). Analysis of the reactivation session data revealed that both vehicle- and garcinol-infused rats exhibited significant and equivalent memory recall during the reactivation session; the ANOVA (group by trial) revealed a significant effect of trial [pre-CS vs. CS; *F*_(1,10)_ = 1091.79, *p*<0.05], but not of group [*F*_(1,10)_ = 0.24; Figure 6b]. Further, as in our previous experiments, we found that intra-LA infusion garcinol had no effect on PR-STM [*t*_(10)_ = 0.58; Figure 6c] but significantly impaired PR-LTM relative to vehicle-infused controls [*t*_(10)_ = 7.74, *p*<0.01; Figure 6c]. Analysis of the neurophysiology revealed that both vehicle- and garcinol-infused rats exhibited significant retention of training-related enhancements in the amplitude of the AEFP in the LA during the PR-STM test relative to baseline [vehicle: *t*_(4)_ = 5.83, *p*<0.05; garcinol: *t*_(6)_ = 5.29, *p*<0.05] that did not differ from each other [*t*_(10)_ = 0.59; Figure 6d]. However, during the PR-LTM test garcinol-treated rats exhibited significantly less AEFP amplitude change relative to vehicle-infused controls [*t*_(10)_ = 4.66, *p*<0.01; Figure 6d]. Thus, intra-LA infusion of garcinol shortly following fear memory retrieval significantly impairs the reconsolidation of a fear memory and, in parallel, leads to a reversal in training-related enhancements in tone-evoked neural activity in the LA.

**Figure 7:**
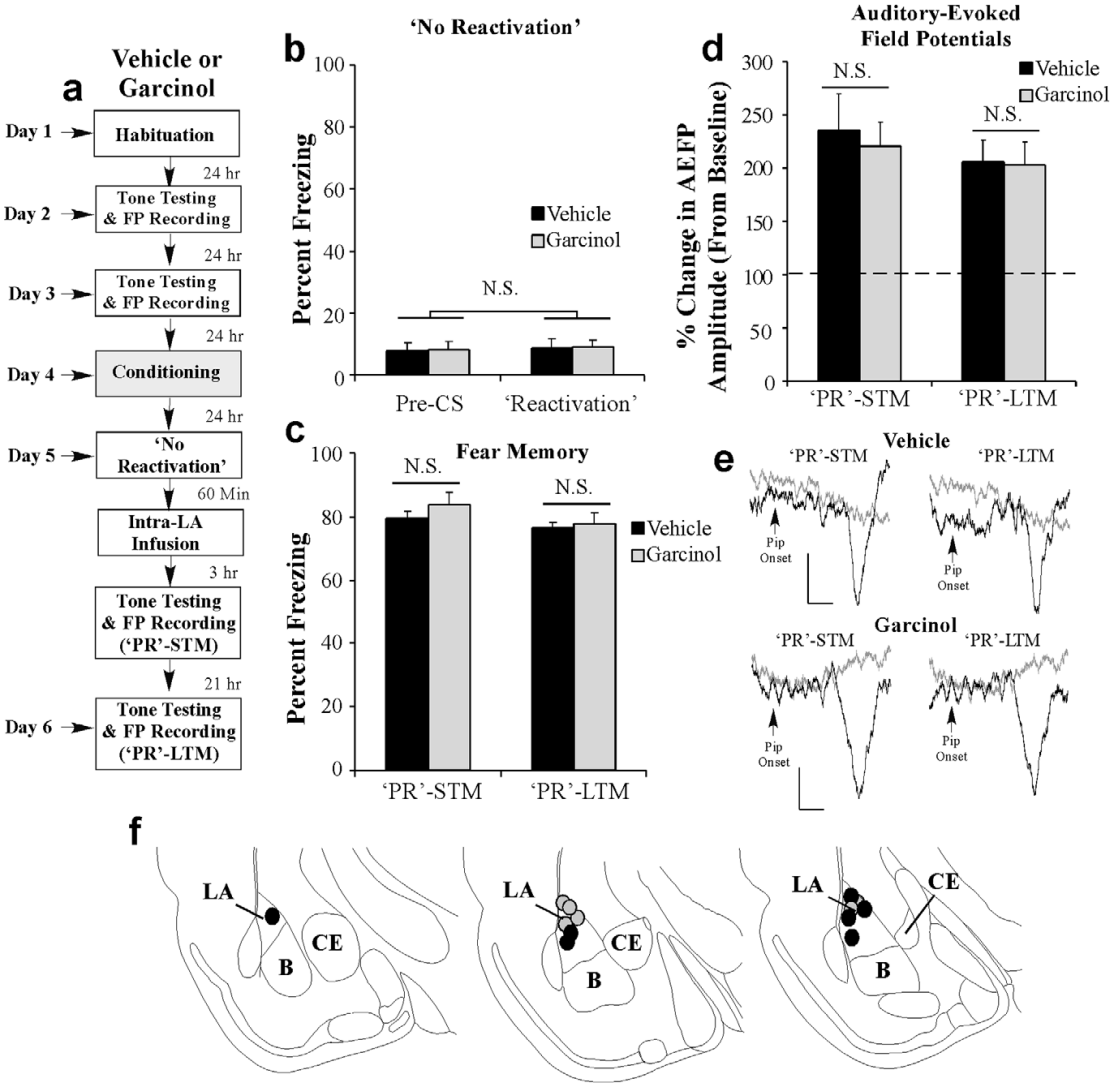
Intra-LA infusion of garcinol in the absence of fear memory retrieval has no effect on fear memory reconsolidation or memory-related neural plasticity in the LA. (a) Rats were given two baseline AEFP recording sessions on separate days followed by fear conditioning with three tone-pip-shock pairings. Twenty four hrs following training rats were given a ‘no-reactivation’ session followed by infusion of vehicle (*n* = 7) or garcinol (500 ng/side; *n* = 6). Rats in each group were then tested for ‘PR’-STM and ‘PR’-LTM 3 and 21 hrs later while AEFPs were recorded from the LA. (b) Memory retrieval data for the vehicle and garcinol-infused groups. (c) Mean (± SEM) percent freezing during the ‘PR’-STM and ‘PR’-LTM tests in vehicle and garcinol-infused groups. (d) Mean (± SEM) percent of change in AEFP amplitude during the ‘PR’-STM and ‘PR’-LTM tests in vehicle and garcinol-infused rats, relative to baseline. *p<0.05 relative to vehicle-infused controls. (e) Representative AEFPs recorded from the LA for each group during baseline (light gray trace), ‘PR’-STM and ‘PR’-LTM sessions (darker traces). Scale bar  = 10 µV, 5 ms. (f) Electrode placements for rats infused with either vehicle (black circles) or garcinol (gray circles).

Importantly, we found that the effect of post-retrieval administration of garcinol on memory-related neural plasticity is specific to active fear memory retrieval. Rats were fear conditioned as before followed 24 h later by a ‘no-reactivation’ session in which they were placed in the testing chamber without a CS presentation. One hr following the ‘no-reactivation’ session, rats received intra-LA infusion of either vehicle (0.5 µl/side) or garcinol (500 ng/side; 0.5 µl) followed 3 and 21 hr later by tests of ‘PR’-STM and ‘PR’-LTM (Figure 7a). As expected, analysis of the ‘no reactivation’ session data revealed that both groups displayed equivalently low levels of freezing during the ‘pre-CS’ period and during the 20 sec period when the CS would have been presented during the reactivation session (Figure 7b). An ANOVA (group by trial) revealed no significant effect of group [*F*_(1,11)_ = 0.04] or trial [*F*_(1,11)_ = 0.11]. Similarly, both vehicle and garcinol-treated rats exhibited equivalently high levels of freezing during the ‘PR’-STM test [*t*_(11)_ = 1.01; Figure 7c] and during the ‘PR’-LTM test [*t*_(11)_ = 0.27; Figure 7c]. Analysis of the neurophysiology revealed that both vehicle and garcinol-infused rats exhibited significant enhancements in AEFP amplitude relative to baseline during the ‘PR’-STM test [vehicle: *t*_(6)_ = 12.20, *p*<0.05; garcinol: *t*_(5)_ = 4.84, *p*<0.05] that did not differ from each other [‘PR’-STM: *t*_(11)_ = 0.33; Figure 7d]. Further, we observed no differences in AEFP amplitude between the two groups during the PR-LTM test [*t*_(11)_ = 0.08; Figure 7d]. Thus, garcinol is only effective at impairing memory-associated neural plasticity when it is administered around the time of active memory recall.

### Systemic administration of garcinol shortly after fear conditioning or fear memory retrieval impairs the consolidation and reconsolidation of a fear memory

Our experiments thus far suggest that local infusion of garcinol into the LA can significantly impair newly formed or reactivated fear memories and associated neural plasticity in the LA. In a clinical setting, however, it is desirable to administer drugs systemically. Accordingly, in our final series of experiments, we asked whether systemic administration of garcinol can impair both fear memory consolidation and reconsolidation.

**Figure 8:**
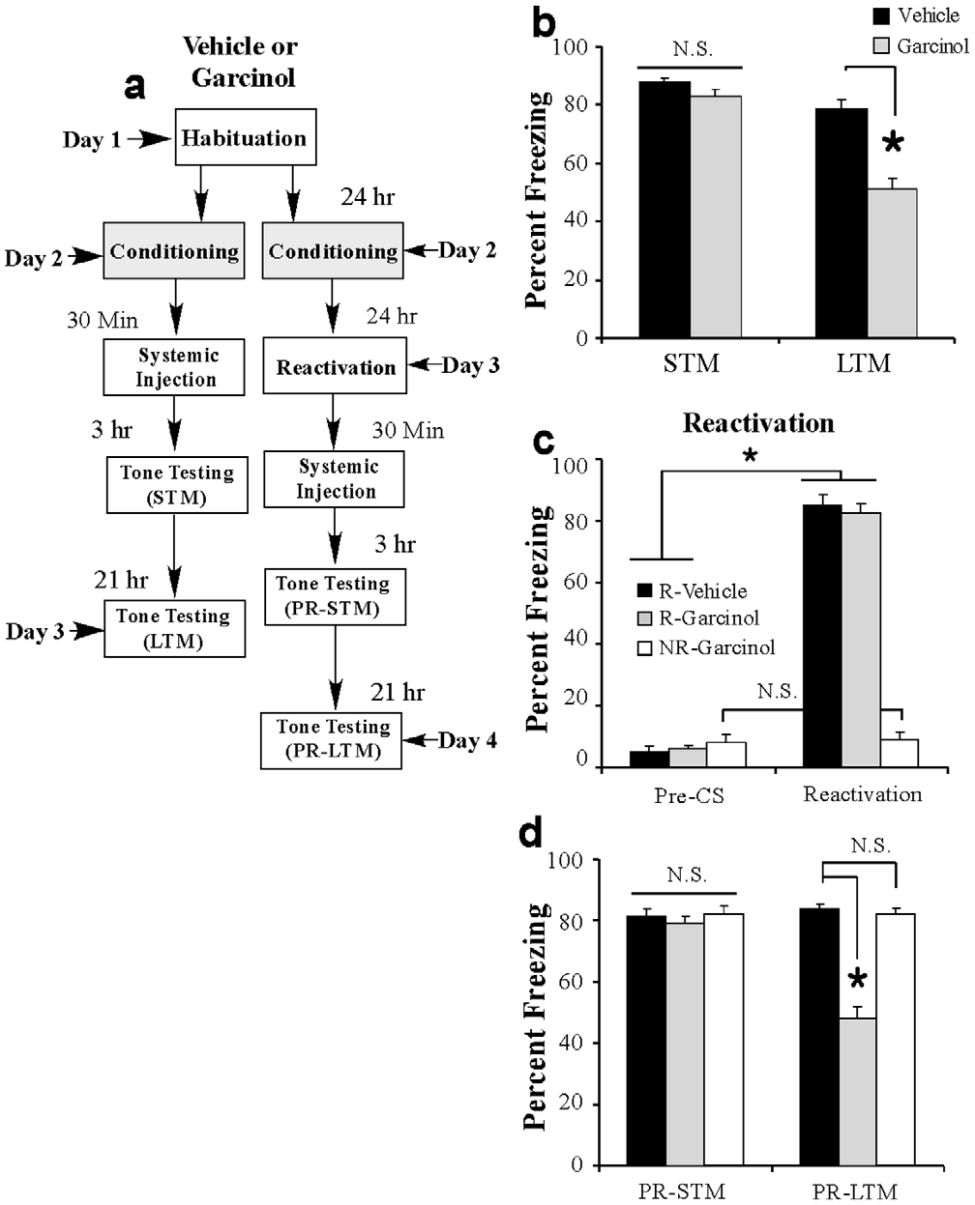
Systemic injection of garcinol impairs the consolidation and reconsolidation of a fear memory. (*a*) Schematic of the behavioral protocol. In the consolidation experiment, rats were fear conditioned with two tone-shock pairings followed 30 min later by i.p. injection of either garcinol (10 mg/kg; *n* = 8) or vehicle (*n* = 8). STM was examined 3 hrs later and LTM 21 hrs following injections. In the reconsolidation experiment, rats were fear conditioned with two tone-shock pairings followed 24 hrs later by fear memory reactivation session and i.p. injection of either garcinol (R-Garcinol; *n* = 9) or vehicle (R-Vehicle; *n* = 9). A third group received garcinol following a no-reactivation control session (NR-Garcinol; *n* = 8). All rats received tests of PR-STM and PR-LTM 3 hrs and 21 hrs after injections, respectively. (b) Mean (± SEM) percent freezing during the STM and LTM tests in vehicle and garcinol-infused groups in the consolidation experiment. * p<0.05 relative to vehicle group. (c) Memory retrieval data for the R-Vehicle, R-Garcinol, and NR-Garcinol groups in the reconsolidation experiment. *p<0.05 relative to the pre-CS period. (d) Mean (± SEM) percent freezing during the PR-STM and PR-LTM tests in R-Vehicle, R-Garcinol, and NR-Garcinol groups. *p<0.05 relative to vehicle-infused controls.

In our first experiment, rats were fear conditioned with two tone-shock pairings. Thirty min following training, rats received i.p. injection of either vehicle or garcinol (10 mg/kg) followed 3 and 21 hr later by tests of STM and LTM, respectively (Figure 8a). Both vehicle and garcinol-treated rats exhibited equivalent levels of freezing during the STM test [*t*_(14)_ = 1.77, *p*>0.05; Figure 8b], indicating, as we observed in our previous experiments, that garcinol does not impair STM. However, the following day garcinol-treated rats exhibited impaired LTM relative to the vehicle-injected group [*t*_(14)_ = 5.86, *p*<0.01; Figure 8b].

In our reconsolidation experiment, rats were fear conditioned as before followed 24 h later by either a fear memory reactivation session or a ‘no-reactivation’ session administered in a distinct context. Thirty min following reactivation, rats received systemic injection of either vehicle or garcinol (10 mg/kg) to comprise three groups: Reactivated (R)-Vehicle, R-Garcinol, and Non-Reactivated (NR)-Garcinol. All three groups were then tested for PR-STM and PR-LTM at 3 and 21 hrs following injection, respectively (Figure 8a). During the reactivation session, both reactivated groups showed significant and equivalent memory recall, while the non-reactivated control group did not (Figure 8c). An ANOVA (group by trial) revealed significant main effects of group [*F*_(2,23)_ = 110.52, *p*<0.01], trial [pre-CS vs. CS; *F*_(1,23)_ = 938.95, *p*<0.01] and the group by trial interaction [*F*_(2,23)_ = 218.93, *p*<0.01]. Duncan's post-hoc t-tests revealed that the R-Vehicle and R-Garcinol groups demonstrated increased freezing during the CS relative to the pre-CS period (*p*<0.05) that was not significantly different from one another (*p*>0.05). We observed no increase in freezing between the ‘pre-CS’ and ‘reactivation’ period in the NR-Garcinol group (*p*>0.05). Further, each of the groups exhibited equivalent levels of memory during the PR-STM test [*F*_(2,23)_ = 0.45; Figure 8d], indicating, as we have observed previously, that garcinol has no effect on the retention of a fear memory shortly after injection. During the PR-LTM test, however, the group injected with garcinol following memory reactivation (R-Garcinol) exhibited impaired PR-LTM relative to the other groups [*F*_(2,23)_ = 63.32, *p*<0.01; Figure 8d]. Duncan's post-hoc t-tests showed that the R-Garcinol group exhibited statistically lower levels of freezing relative to both the R-Vehicle and NR-Garcinol groups (*p*<0.01), which were not found to differ from one another (*p*>0.05).

## Discussion

While the study of the cellular and molecular mechanisms underlying the consolidation and reconsolidation of traumatic fear memories has attracted considerable experimental interest [5]–[10], few compounds have to date emerged that are readily useful in a clinical setting. Recent studies, however, have suggested that the targeting of ‘epigenetic’ processes, including modifications in chromatin structure and function, may hold considerable promise in the treatment of neuropsychiatric diseases that affect memory and cognition [49]–[53]. In this study, we have systematically investigated the potential efficacy of garcinol, a naturally-occurring HAT inhibitor derived from the diet, in mitigating the consolidation and reconsolidation of Pavlovian fear memories, a type of persistent aversive memory that is characteristic of anxiety disorders such as PTSD [4]. We show that local infusion of garcinol into the LA, the presumed locus of storage of fear memories [64], impairs the training and retrieval-related acetylation of histone H3 in the LA. We further show that intra-LA or systemic administration of garcinol within a narrow window after either fear conditioning or fear memory recall, respectively, significantly impairs the consolidation and reconsolidation of a Pavlovian fear memory and associated neural plasticity in the LA.

Garcinol is a polyisoprenylated benzophenone compound extracted from the rind of the fruit of *Garcinia indica*, also known as Kokum, a tree native to the tropical coastal regions of Western India [65], [66]. While typically not eaten as a fresh fruit, Kokum rind is instead frequently dried and used as a seasoning for curries or processed into a syrup suitable for drinking [66]. The readily consumable juice made from the rind of the Kokum fruit has been prevalently used in Ayurvedic medicine to treat a remarkably wide range of ailments, including inflammation, infection, dermatitis, and gastrointestinal problems [66]. Empirical studies have further identified anti-oxidant, anti-obesity, anti-tumor and anti-inflammatory actions of garcinol or its derivatives [65]–[69]. While there are over a dozen existing patents for the potential efficacy of garcinol in the treatment of various conditions ranging from inflammation to obesity to cancer [65], our findings are the first to suggest that garcinol may also be effective, either alone in combination with existing pharmacological or behavioral interventions, in the treatment of neuropsychiatric disorders such as PTSD. Future experiments will be necessary to evaluate this possibility.

At the molecular level, garcinol has been shown to be a potent inhibitor of the HAT activity of CREB-binding protein (CBP), E1A-associated protein (p300), and the p300/CBP-associated factor (PCAF) [55], [70]. Each of these HATs has been widely studied in memory formation and synaptic plasticity, most notably using molecular genetic approaches with a focus on hippocampal-dependent memory paradigms including object recognition, spatial memory and contextual fear memory [71]–[81]. These studies have complemented existing pharmacological studies that have implicated HAT and HDAC activity in hippocampal long-term potentiation (LTP) and hippocampal-dependent memory [75], [82]–[89]. To date, however, only two studies have implicated HATs in amygdala-dependent ‘cued’ fear memory formation in a genetically modified mouse model [49], [90] while most have found no effect [71]–[74], [79], [81]. These findings suggest that many of the existing mouse molecular genetic models may not be optimal to reveal a role for HATs in amygdala-dependent memory. In contrast, we have shown in the rat that auditory fear conditioning is associated with an increase in the acetylation of histone H3, but not H4, in the LA [58], and that intra-LA infusion of the HDAC inhibitor TSA enhances both H3 acetylation and the consolidation of an auditory fear memory; that is, STM is not affected, while LTM is significantly enhanced [58]. Further, bath application of TSA to amygdala slices significantly enhances LTP at thalamic and cortical inputs to the LA [58]. Consistent with these findings, in the present study we show that intra-LA infusion of the HAT inhibitor garcinol significantly impairs training-related H3 acetylation and the consolidation of an auditory fear memory and associated neural plasticity in the LA; STM and short-term enhancements in tone-evoked neural activity in the LA are intact, while LTM and long-term training-related neural plasticity are significantly impaired. Collectively, our findings point to an important role for chromatin modifications in the consolidation of amygdala-dependent fear memories. Additional experiments will be required to examine the specific HATs that are targeted by garcinol after fear conditioning and the mechanisms by which they promote fear memory consolidation and long-term alterations in synaptic plasticity in the LA.

This is the first study, of which we are aware, to systematically examine the role of a pharmacological inhibitor of HAT activity in memory reconsolidation processes. We show that intra-LA infusion of garcinol following auditory fear memory retrieval impairs retrieval-related histone H3 acetylation in the LA and significantly interferes with the reconsolidation of a fear memory and that of memory-related neural plasticity in the LA; that is, PR-STM and associated neural plasticity are unaffected, while PR-LTM is impaired together with a loss of memory-related plasticity in the LA. We further show that the effect of garcinol on memory reconsolidation and memory-associated plasticity in the LA is specific to a reactivated memory and temporally restricted; we observed no effect of garcinol in the absence of memory reactivation or following a delayed infusion, findings which rule out the possibility that garcinol, at the doses chosen here, may have damaged the amygdala or produced other non-specific effects that may have affected the reconsolidation process. Importantly, post-retrieval treatment with garcinol was observed to effectively impair the reconsolidation of both a recently formed (within 24 hrs) and a ‘well-consolidated’ (2 week old) fear memory, suggesting that even older fear memories are susceptible to reconsolidation impairment using this compound. This latter finding adds to a growing body of evidence that amygdala-dependent memories are susceptible to reconsolidation interference regardless of their age [19], [25], [33], and has important implications for the use of reconsolidation-based approaches in a clinical setting. Finally, and perhaps most importantly, we show that fear memories that fail to reconsolidate following post-retrieval treatment with garcinol are lost in an enduring manner; they are not subject to spontaneous recovery, to reinstatement following a series of unsignaled footshocks, or to a shift in the testing context, all trademark characteristics of fear memories that are lost due to fear extinction or exposure-based procedures [91]-[93]. This latter finding is particularly important not only in a clinical context, but it also rules out the possibility that garcinol may have influenced fear memory reconsolidation processes by promoting facilitated extinction after the reactivation trial. Indeed, a recent report has suggested that infusion of a p300-specific HAT inhibitor into the prefrontal cortex can paradoxically enhance fear extinction [94]. Our findings, in contrast, suggest that fear extinction has not been enhanced by garcinol; rather, local infusion of garcinol into the LA appears to have specifically interfered with fear memory reconsolidation.

In summary, our findings provide strong evidence that a naturally-occurring HAT inhibitor derived from the diet can significantly impair either newly formed or reactivated fear memories in a widely studied animal model of PTSD. Our findings suggest that garcinol and other yet to be identified compounds that target the regulation of chromatin function or structure may hold great promise as therapeutic agents in alleviating fear and anxiety disorders characterized by persistent, unwanted memories when administered either shortly after traumatic memory formation or in conjunction with ‘reconsolidation’ based forms of psychotherapy.

## Materials and Methods

### Subjects

Adult-male Sprague-Dawley rats (Harlan), weighing 300–350 g and aged 2–3 months, were housed individually in plastic cages and maintained on a 12∶12 hr light/dark cycle with food and water provided *ad libitum*.

### Surgery

Rats were anesthetized with i.p. administration of Ketamine (100 mg/kg) and Xylazine (6.0 mg/kg) and implanted with 26-gauge stainless-steel guide cannulas (Plastics One, Roanoke, VA) in the LA (−3.2 mm, ±5.2 mm, −8.0 mm relative to Bregma). Guide cannulas were secured to screws in the skull using a mixture of dental acrylic and cement and 31-gauge dummy cannulas were inserted into the guide to prevent obstruction. Buprenex (0.2 mg/kg) was administered as an analgesic and rats were provided with at least five days post-operative recovery time. All surgical procedures were conducted under the guidelines provided in the National Institutes of Health *Guide for the Care and Use of Experimental Rats* and were approved by the Yale University Institutional Animal Care and Use Committee.

### Electrode implantation procedures

Rats were anesthetized under the same procedures as those used for cannula implantation. Rats were implanted in the left LA with a tungsten recording electrode (0.1 mm diameter, 1 MΩ) adhered to a 26-gauge guide cannula (AP: −3.2 mm; ML: ±5.2; DV: −7.4). The recording wire extended 0.75 mm beyond the base of the guide. A 26-gauge guide cannula was implanted in the right-LA. A low-impedance copper wire was connected to a stainless steel bone screw drilled into the skull contralateral to the side of the recording electrode ∼1 mm posterior to Bregma to serve as the reference for recording purposes. Another stainless steel screw attached to a copper wire was drilled into the skull ∼3 mm posterior to lambda and served as the ground electrode. Dental cement was used to anchor the electrodes and connecting device to the skull. Rats were given at least 5 days to recover from the surgery before experiments.

### Drugs

The PCAF/p300 HAT inhibitor garcinol (Enzo; BML-GR343) was dissolved in 100% DMSO to a 2 µg/µl stock solution and then diluted 1∶1 in ACSF to a final concentration of 1 µg/µl prior to infusion into the brain. The vehicle solution for intra-cranial infusion experiments consisted of 50% DMSO. For systemic experiments, garcinol was dissolved in 100% DMSO to a stock solution of 10 mg/mL and administered i.p. at a 10 mg/kg dose. Vehicle solution for systemic experiments consisted of 100% DMSO.

### Pharmacology and Western blotting experiments

We have recently shown that auditory Pavlovian fear conditioning, but not exposure to tone or shocks alone, leads to an increase in the acetylation of histone H3 in the LA that is most prominent at 90 mins after fear conditioning [58]. In a related study, we showed that auditory fear memory retrieval, but not exposure to tone alone or to the context in the absence of fear memory reactivation, leads to a similar increase in histone H3 acetylation at 90 mins [59]. To examine the effect of garcinol on fear conditioning-related histone acetylation in the LA, cannulated rats were habituated to handling and the conditioning chambers (30 min/day/chamber) for four days prior to auditory fear conditioning consisting of three tone-shock pairings (30 sec, 5 kHz, 75 dB; 1.0 mA). The conditioning chamber (Chamber A) was a lit chamber with a grid floor. One-hr after tone-shock pairings rats were infused with either vehicle (0.5 µl/side) or garcinol (500 ng/side; 0.5 µl). Thirty-min later (90 min following training) rats were given an overdose of chloral hydrate (600 mg/kg; i.p.) and brains were removed and frozen at −80°C. An additional group of naive rats was handled and habituated but not exposed to the training chamber prior to infusion of 50% DMSO vehicle (0.5 µl/side) and was sacrificed 30 min following infusions. To examine the effect of garcinol on fear memory retrieval-related histone acetylation in the LA, rats were habituated to both the conditioning (Chamber A) and testing chambers for four days. The testing chamber (Chamber B) consisted of a dark chamber with a black plastic floor which was washed immediately before the reactivation session with a distinctive peppermint soap. On the fifth day, rats were given three tone-shock pairings in Chamber A. The next day, rats were given an auditory fear memory reactivation session consisting of a single presentation of a 30 sec, 5 kHz, 75 dB tone administered in Chamber B. One hour later, rats were given intra-LA infusions of either vehicle (0.5 µl/side) or garcinol (500 ng/side; 0.5 µl). Thirty min later (90 min after the reactivation session) all rats were given an overdose of chloral hydrate (600 mg/kg; i.p.), and brains were removed and frozen at −80°C.

Punches containing the LA around the cannula tips were obtained with a 1 mm punch tool (Fine Science Tools, Foster City, CA) from 400-µm-thick sections taken on a sliding freezing microtome. Punches were manually dounced in 100 µl of ice-cold hypotonic lysis buffer [10 mM Tris-HCl, pH 7.5, 1 mM EDTA, 2.5 mM sodium pyrophosphate, 1 mM phenylmethylsulfonyl fluoride, 1 mM β-glycerophosphate, 1% Igepal CA-630, 1% protease inhibitor cocktail (Sigma) and 1 mM sodium orthovanadate]. Sample buffer was immediately added to the homogenates, and the samples were boiled for 4 min. Homogenates were electrophoresed on 18% Tris-HCl gels and blotted to Immobilon-P (Millipore, Bedford, MA). Western blots were then blocked in TTBS buffer (50 mM Tris-HCl, pH 7.5, 150 mM NaCl, and 0.05% Tween-20) with 5% dry milk and then incubated with the appropriate primary antibody [AcH3 (pan), 1∶3,000, Millipore; total H3, 1∶5,000, Millipore]. Blots were then incubated with anti-rabbit antibody conjugated to horseradish peroxidase (Cell Signaling, Danvers, MA) and developed using West Dura chemiluminescent substrate (Pierce Laboratories, Rockford, IL). Western blots were developed in the linear range used for densitometry. Densitometry was conducted using Image J software. To control for inconsistencies in loading, optical densities for total H3 protein were first normalized to GAPDH protein (1:20,000; Abcam). Acetylated H3 protein was then normalized to total H3 protein. For analysis, all data were normalized to the average value of naïve controls and analyzed using ANOVA.

### Behavioral experiments

Rats were handled for two days prior to conditioning. On the second handling day, dummy cannulas were removed to check for patency. Rats were then habituated to Chamber A for 15 minutes (Day 1). The following day (Day 2), rats were placed in Chamber A and presented with three tone-shock pairings consisting of a 30 sec, 5 kHz, 75 dB tone that co-terminated with a 1 sec, 1.0 mA foot shock. One hour later, rats received intra-LA infusion of either vehicle (0.5 µl/side) or garcinol (500 ng/side; 0.5 µl). Infusions were made over 4 min and the infusion cannulas were left in place for at least 2 min following infusion to facilitate diffusion throughout the LA. Three hr after infusions, rats were tested for short-term memory (STM) consisting of the presentation of three CS tones (30 sec, 5 kHz, 75 dB) in Context B. Twenty-one hr later (Day 3), all rats received a long-term memory (LTM) test consisting of 10 tone CS presentations (30 sec, 5 kHz, 75 dB) in context B.

For the reconsolidation experiments, rats were habituated and conditioned as before. The next day (Day 3), rats were placed in Chamber B and received either a single tone CS presentation, to serve as a memory reactivation trial, or no tone presentation, to serve as a ‘no reactivation’ trial. One hour later, rats received intra-LA infusion of either vehicle (0.5 µl/side) or garcinol (500 ng/side; 0.5 µl). Three hr after infusions, rats were tested for post-reactivation short-term memory (PR-STM) consisting of the presentation of three CS tones (30 sec, 5 kHz, 75 dB) in Context B. Twenty-one hr later (Day 3), all rats received a post-reactivation long-term memory (PR-LTM) test, which consisted of 10 tone CS presentations (30 sec, 5 kHz, 75 dB) in context B. Rats used to examine the effect of HAT inhibition on the reconsolidation of a “well-consolidated” memory were tested under identical parameters, however they were returned to their homecage for two weeks following conditioning prior to the reactivation session.

An additional behavioral experiment examined whether the reconsolidation deficit induced by HAT inhibition in the LA was sensitive to spontaneous recovery, reinstatement, or to a shift in the testing context. The protocol for this experiment was adapted from that of a previous study by Duvarci and Nader [60]. Rats in this experiment were trained in Chamber A, reactivated 24 hrs later in Chamber B and given intra-LA infusion of vehicle or garcinol as described above. Three and 21 hr after infusion, rats were returned to Chamber B and tested for PR-STM and PR-LTM, respectively. One week after the initial PR-LTM test rats were returned to Chamber B and tested for spontaneous recovery with five tone CS presentations. The next day, they were placed in a novel context (Chamber C), scented with cedar and brightly illuminated, and given a reinstatement session consisting of three unsignaled footshocks (1 sec, 1.0 mA). Twenty-four hours later, all rats were returned to Chamber B and tested for reinstatement of fear with five tone CS presentations. The next day, rats were introduced to a final novel context (Chamber D), consisting of a lit behavior box with a floral scented cotton-padded floor, and tested with three tone CS presentations to examine the context generality of the reconsolidation deficit.

Behavioral experiments employing systemic garcinol injections were conducted using non-cannulated rats, and, accordingly, a slightly weaker fear conditioning paradigm was used consisting of 2 tone-shock pairings (1 sec, 0.5 mA). Thirty-min after conditioning, rats received i.p. injection of either vehicle or garcinol (10 mg/kg). Here, we used a 30 min post-training injection time point (rather than 1 hr as in our intra-LA experiments) to allow additional time for the drug to enter the system. STM and LTM were examined at 3 and 21 hr following injections in Chamber B. Examination of the effect of systemic garcinol administration on fear memory reconsolidation was conducted under the aforementioned parameters, however twenty-four hr after training rats were given either a tone-reactivation or no-reactivation session followed by i.p. injections 30 min later and subsequent PR-STM and PR-LTM tests.

Each behavioral test was videotaped for subsequent scoring and scored by an observer who was blind to the experimental conditions. Freezing was defined as a lack of movement, excluding that necessary for respiration, and was quantified as a percentage of the amount of time the rat spent engaged in freezing behavior during the CS presentations. All data were analyzed with ANOVA and Duncan's post-hoc *t*-tests. Repeated measures ANOVAs were used for multiple trial comparisons. Differences were considered significant if *p*<0.05. Only data from those rats with bilaterally well-placed cannulas within the borders of the LA were included in the analyses.

### Neurophysiological recordings

Awake-behaving neurophysiology took place in a custom-made electromagnetic shielded recording chamber designed for delivery of auditory stimuli and recording. The chamber was kept within a ventilated and temperature-regulated acoustic isolation room. Stimulus delivery and data acquisition were controlled by SciWorks Experimenter Real-time 7.0 (DataWave). During recording, rats were exposed to a modified CS consisting of a series of tone ‘pips’ (20 presentations of a 50 ms, 75 dB, 1 kHz tone pips, delivered at a frequency of 1 Hz) from a speaker mounted on the ceiling of the recording chamber. The tone pips were triggered by TTL signals generated by SciWorks. The TTL signals were converted (Coulbourn, H91-24, 5 V TTL to 24 V converter) and sent to a tone generator (Coulbourn, H12-07, Seven-Tone Audio Cue). During recordings, the implanted electrodes were connected to a Micro-Miniature Headstage (DataWave). Neural signals were picked up (Legacy PCI data acquisition bundles, Model: DT3010), amplified (16-channel A-M Systems microelectrode amplifier, Model: AM-3600) and saved for off-line analysis.

On day 1 of each experiment, rats were handled and habituated to the recording chamber and cable connection for 15 min each. On days 2 and 3, baseline auditory-evoked field potentials (AEFPs) elicited by 3 presentations of the 20 tone-pip CS series were recorded (ITI  = 2 mins) from the LA, for a total of 60 tone pip presentations. On day 4, rats received 3 tone-pip shock pairings in an illuminated chamber consisting of a series of 20-tone-pip presentations which co-terminated with a 1s, 1.0mA footshock administered through the grid-floor. One-hr following training rats received intra-LA infusion of either vehicle (0.5 µl/side) or garcinol (500 ng/side; 0.5 µl). Three-hrs later rats were placed into a modified chamber which included a flat black peppermint scented floor for STM testing and AEFP recordings consisting of 3 presentations of the tone-pip CS series were recorded (ITI  = 2 mins), for a total of 60 tone pip presentations (identical to baseline recordings). The following day rats were placed back in the modified chamber and examined for LTM with 9 tone-pip presentations.

For the reconsolidation experiments, rats underwent habituation, baseline recording sessions, and fear conditioning as in the consolidation experiment. The next day (Day 5), rats were placed in the modified chamber (black peppermint scented floor) and received either a single tone-pip series presentation, to serve as a memory reactivation trial, or no tone-pip presentation, to serve as a ‘no-reactivation’ trial. One-hour later rats were infused with either vehicle (0.5 µl/side) or garcinol (500 ng/side; 0.5 µl). Three-hrs after infusions, rats were tested for PR-STM and AEFPs with 3 presentations of the tone-pip CS series, in the modified chamber. Twenty-one hr later (Day 6) rats were tested for PR-LTM and AEFPs with 9 presentations of the tone-pip CS series.

Rats' freezing behavior was recording during all sessions for off-line scoring. Following the completion of testing all rats were rapidly and deeply anesthetized prior to transcardial-perfusions and brain extractions for electrode placement analyses. For data analysis during STM/PR-STM sessions, all 60 AEFPs were averaged into a single waveform. Data analysis for the LTM/PR-LTM sessions was conducted based on the average waveform from the last 60 AEFPs. Spike2 software (Cambridge Electronics Design, Cambridge, UK) was used to analyze the amplitude of the short-latency negative-going component of the AEFP from the initial point of deflection to its maximal negativity, which occurs ∼12–16 ms from the onset of the pip [62]–[64]. The amplitude of AEFPs recorded during the STM and LTM tests were expressed as a percentage of the baseline amplitude for comparison between vehicle and garcinol-treated groups. Data were analyzed using t-tests and differences were only considered significant is *p*<0.05.
