# Correction: A Naturally-Occurring Histone Acetyltransferase Inhibitor Derived from *Garcinia indica* Impairs Newly Acquired and Reactivated Fear Memories

**DOI:** 10.1371/journal.pone.0181892

**Published:** 2017-07-17

**Authors:** Stephanie A. Maddox, Casey S. Watts, Valérie Doyère, Glenn E. Schafe

The authors would like to clarify that this study was generated from the same large data set as another study published by three of the same authors, titled “p300/CBP histone acetyltransferase activity is required for newly acquired and reactivated fear memories in the lateral amygdala”. The two publications present experiments which were performed concurrently. The authors ran Western blotting, behavioral and neurophysiological experiments for vehicle, garcinol and c646 at the same time. The same vehicle control group is represented in both papers in the behavioral and neurophysiology experiments. The Western blot experiments in each paper were run with separate vehicle controls that were injected and sacrificed on the same day as the garcinol or c646-infused rats.
